# Soft X-ray wavefront sensing at an ellipsoidal mirror shell

**DOI:** 10.1107/S1600577524003643

**Published:** 2024-06-06

**Authors:** Christoph Braig, Jürgen Probst, Heike Löchel, Ladislav Pina, Thomas Krist, Christian Seifert

**Affiliations:** a Institute of Applied Photonics e.V., Rudower Chaussee 29/31, 12489Berlin, Germany; b NOB Nano Optics Berlin GmbH, Krumme Straße 64, 10627Berlin, Germany; c Czech Technical University Brehova 7 115 19Prague 1 Czech Republic; European XFEL, Germany

**Keywords:** X-ray optics, ellipsoidal mirror, wavefront sensing, focus reconstruction, surface characterization, slope error

## Abstract

A reliable ‘*in situ*’ method for wavefront sensing in the soft X-ray domain is reported, developed for the characterization of rotationally symmetric optical elements, like an ellipsoidal mirror shell. In a laboratory setup, the mirror sample is irradiated by an electron-excited (4.4 keV), micrometre-sized (∼2 µm) fluorescence source (carbon *K*_α_, 277 eV). Substantially, the three-dimensional intensity distribution *I*(**r**) is recorded by a CCD camera (2048 × 512 pixels of 13.5 µm) at two positions along the optical axis, symmetrically displaced by ±21–25% from the focus. The transport-of-intensity equation is interpreted in a geometrical sense from plane to plane and implemented as a ray tracing code, to retrieve the phase Φ(**r**) from the radial intensity gradient on a sub-pixel scale. For reasons of statistical reliability, five intra-/extra-focal CCD image pairs are evaluated and averaged to an annular two-dimensional map of the wavefront error . In units of the test wavelength (C *K*_α_), an r.m.s. value  = ±10.9λ_0_ and a peak-to-valley amplitude of ±31.3λ_0_ are obtained. By means of the wavefront, the focus is first reconstructed with a result for its diameter of 38.4 µm, close to the direct experimental observation of 39.4 µm (FWHM). Secondly, figure and slope errors of the ellipsoid are characterized with an average of ±1.14 µm and ±8.8 arcsec (r.m.s.), respectively, the latter in reasonable agreement with the measured focal intensity distribution. The findings enable, amongst others, the precise alignment of axisymmetric X-ray mirrors or the design of a wavefront corrector for high-resolution X-ray science.

## Abstract 2

Phase retrieval at an axisymmetric ellipsoidal mirror is demonstrated with soft X-rays (277 eV), completed by a reconstruction of the focus as well as figure and alignment error of the mirror. Based on simple measurements of the cross-focal, three-dimensional intensity distribution, an intuitive algorithm is proposed which combines the advantages of high resolution, sensitivity and reliability even under difficult conditions like a low source flux or weak spatial coherence.

## 1. Introduction

The ellipsoidal mirror of rotational symmetry plays an important role in soft X-ray science, such as in microscopy (Müller *et al.*, 2014 ▸; Nawaz *et al.*, 2015 ▸). As an achromatic optical element of large numerical aperture (NA), it may provide efficient point-to-point focusing of weak laboratory sources with an often broadband spectral emission (Holburg *et al.*, 2019 ▸) and in ‘photon hungry’ experiments at beamlines. Applications both in scanning microscopy (Kördel *et al.*, 2020 ▸) and spectroscopy benefit from high resolution, *i.e.* at best, a nearly diffraction-limited focal spot size. This aim is, in particular, enabled by a precisely replicated (Chon *et al.*, 2006 ▸; Kume *et al.*, 2019 ▸) and adjusted mirror, namely a well known figure/alignment and slope error of low magnitude. Obviously, the closed shape and small size of laboratory-scaled ellipsoidal mirror shells precludes the metrology of the inner surface of the mirror with established techniques like long-trace (Siewert *et al.*, 2012 ▸) or interferometric (Kühnel *et al.*, 2021 ▸) profiling. Instead, phase retrieval methods such as grating interferometry (Wang *et al.*, 2013 ▸; Kayser *et al.*, 2017 ▸), ptychography (Takeo *et al.*, 2020 ▸) or speckle correlation analysis (Kim *et al.*, 2017 ▸) are being used. However, the requirements on coherence and the experimental effort give reasons for ‘easy to use’ alternatives like the (Shack–)Hartmann (Keitel *et al.*, 2016 ▸) or coded mask (Wang *et al.*, 2017 ▸) sensor, for instance – supplemented by machine learning, where applicable (Nishizaki *et al.*, 2019 ▸; Qiao *et al.*, 2021 ▸). Unfortunately, even those modern concepts still suffer from a limited spatial resolution or absorption loss in the hole/microlens array or binary transmission plate, respectively.

In this paper, we present a simple and robust approach to maskless, CCD-based wavefront sensing at axisymmetric extreme ultraviolet (XUV) and X-ray optics with an annular aperture, as an extension of our recently developed concept for one-dimensional (1-D) focusing, curved mirror segments of spherical shape (Probst *et al.*, 2020*a* ▸). In Section 2, we specify the optic under test and describe the experimental setup. Under opposite defocus, pairs of intensity patterns, recorded by a CCD camera, are used for the phase retrieval in Section 3. The focus and the combined figure/alignment as well as slope error are reconstructed in Section 4, and the results are compared with data from direct focus measurements. Section 5 concludes with a discussion of the principle and an outlook to potential improvements.

## 2. Optical setup and ellipsoidal mirror specification

Excited by an electron beam at an acceleration voltage of about 4.4 keV (Jeol 6400), C *K*_α_ fluorescence is induced at an energy of 277 eV from a carbon (HOPG) target (Probst *et al.*, 2020*b* ▸), slightly contaminated with bremsstrahlung and minor contributions from O *K*_α_ at 525 eV, due to surface oxidation.^1^ For a sufficiently low e^−^ current of a few μA, the almost point-like, nonetheless incoherent, soft X-ray source with an estimated diameter of ∼2 µm (Gaussian full width at half-maximum, FWHM) emits an approximately spherical wavefront towards the ellipsoidal optic under test.

**Figure 1:**
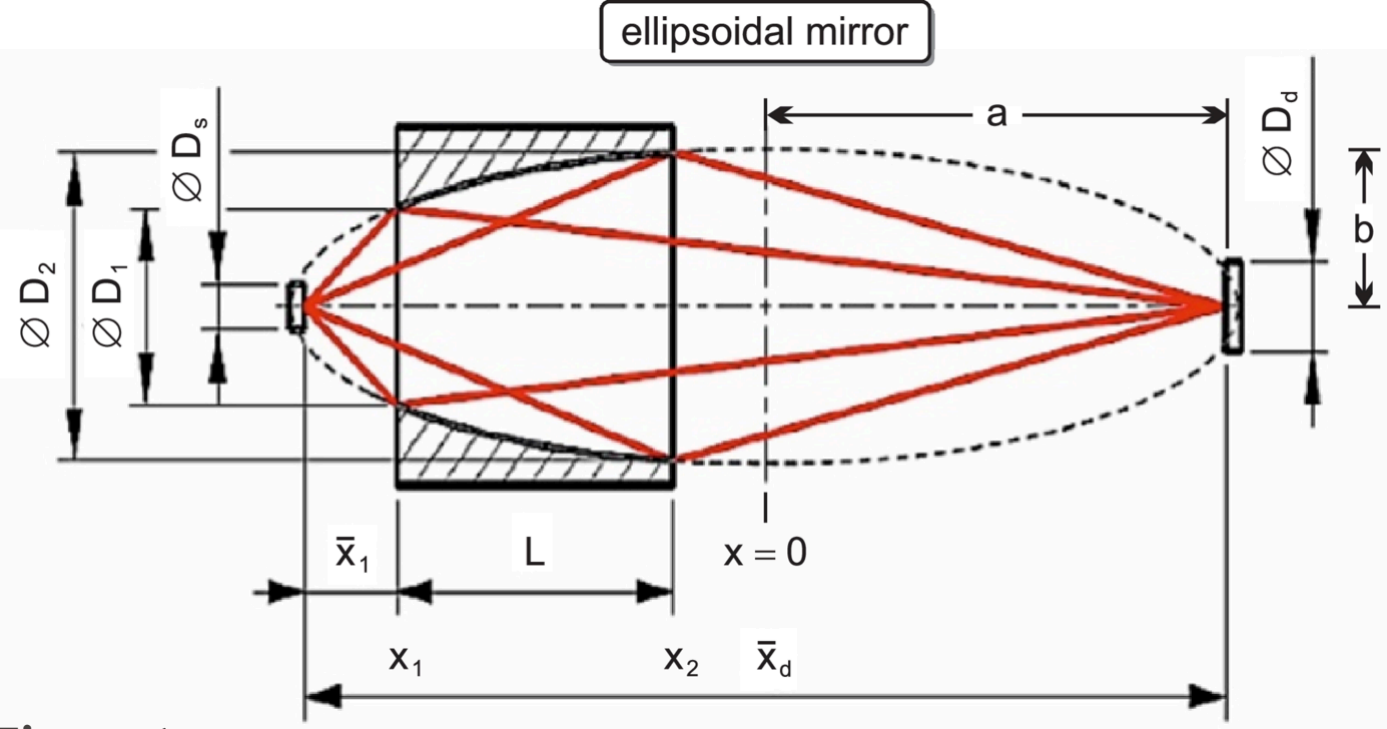
Dimensions of the ellipsoid (dashed curve), defined by its semi-major and -minor half axes, *a* and *b*, respectively. The hatched region represents the mirror section in use, characterized by entrance separation 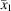, length *L* and focal distance 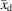, with 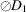 and 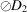 as the inner and outer aperture of the mirror, respectively. 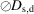 symbolize the source and focus diameter (FWHM). Marginal rays are drawn in red. The notation refers to Table 1 ▸, and the graphic (not to scale) is adopted from https://www.rigaku.com.

The mirror shell of rotational symmetry (Pína, 2019 ▸), formed from a mandrel (Romaine *et al.*, 2009 ▸; Arcangeli *et al.*, 2017 ▸; Yamaguchi *et al.*, 2020 ▸), is realized as an off-centred section of an ellipsoid, defined by its semi-major axis *a* and the – much smaller – semi-minor axis *b*, as sketched in Fig. 1 ▸. For the source in the left of the two ellipsoidal foci at *x* = ±*e*, the radius 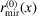 of the ideal mirror is given as 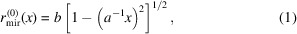
and the excentricity
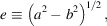
halves the focal distance, 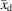 = 2*e*. With an entrance distance 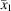 and mirror length *L*, the aperture *D*_1,2_ yields an acceptance solid angle of 2.2 × 10^−3^ sterad. Under grazing incidence at an angle 〈θ〉_*x*_ = 1.23 ± 0.06°, the Au coating reflects 76.2 ± 2.6% at an energy of 277 eV, where a diffractive (Aschenbach, 2008 ▸) microroughness of ±1 nm (r.m.s.) is assumed in the simulation (https://henke.lbl.gov), in agreement with ‘typical’ values of ‘0.3–2 nm’ (https://www.rigaku.com/products/optics/replicated) for such mirrors. Mounted in an optical holder with orthogonal lever arms of 10 cm, the mirror can be aligned manually in its two-dimensional (2-D) angular orientation utilizing micrometre screws (Feinmess Suhl GmbH) with a nominal increment of 10 µm, corresponding to an accuracy of ±10 arcsec – or less, by sensitive handling. The distance 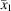 between source and entrance aperture is adjusted on a linear translation stage with a similar precision of at least ±5 µm.

Neglecting off-axis aberrations, the small source of 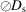 in size is focused to a spot of 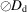 in the image plane at *x* = +*e*. The magnification 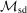

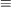

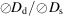
 follows from the geometry in Fig. 1 ▸ approximately as 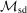

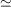

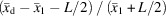
. With 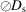 = 2.0 µm and the parameters from Table 1 ▸, we expect a focus size 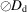 = 7.3 µm. A CCD camera (greateyes ALEX-s 2048 × 515 BI UV1) with a pixel size 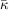 = 13.5 µm and an 18-bit dynamic range is placed on an optical bench at a variable distance 250 mm ≤ *x* ≤ 750 mm from the centre. The whole setup is evacuated to 10^−5^ mbar.

## 3. Measurements under defocus and phase retrieval

**Figure 2:**
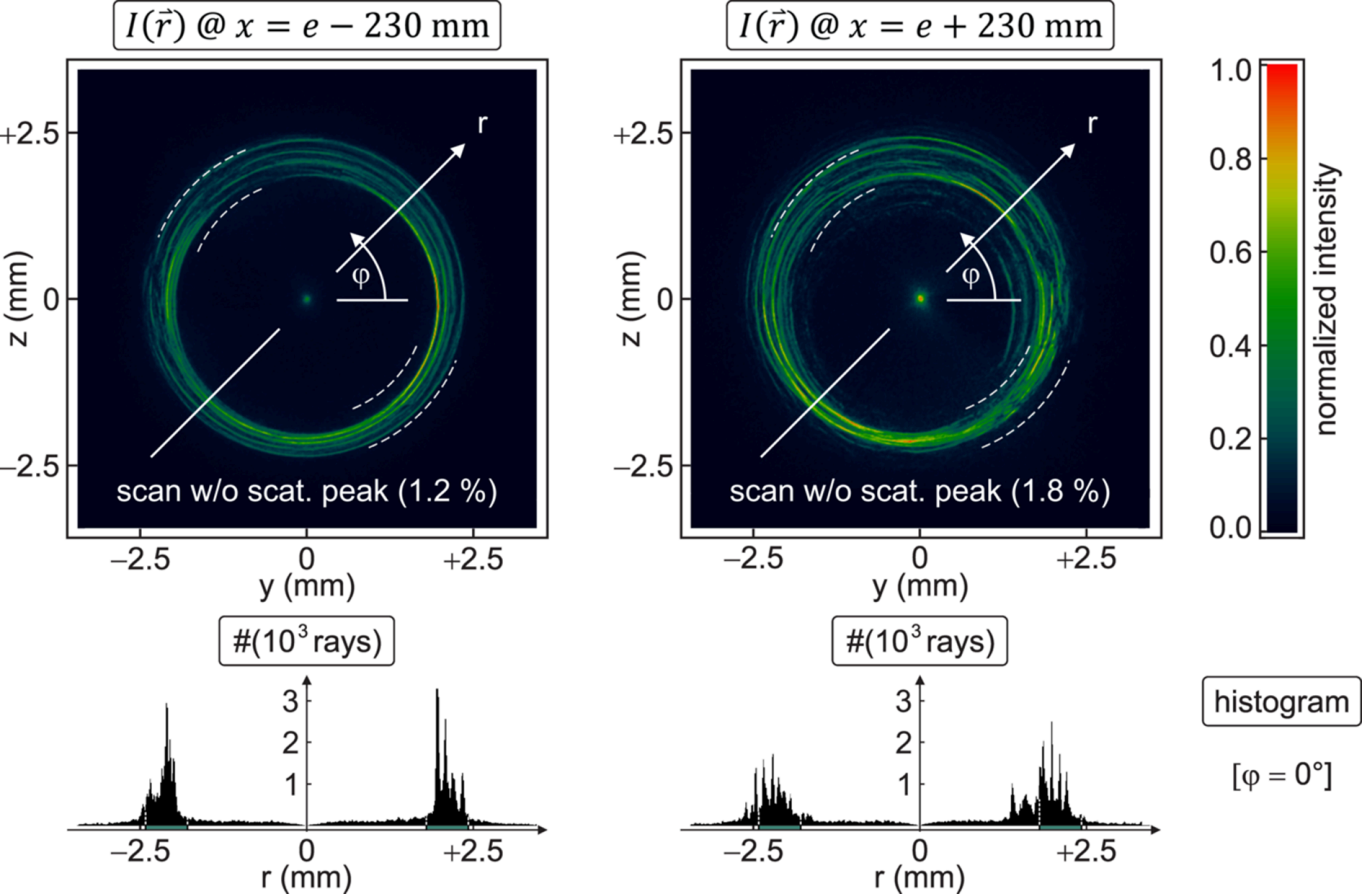
Representative examples of the normalized intensity distribution *I*(**r**), recorded with the CCD camera at two opposite intra- and extra-focal positions in a defocus of −230 mm and +230 mm (top). The central scatter peak, initially used to align the patterns, contributes ∼1–2% to the total power and is erased from all data sets before phase retrieval. Each image is composed of 512 × 512  pixels (13.5 µm) and scanned in the radial direction *r* at angles 0° ≤ φ < 180°. White dashed arcs indicate the expected geometrical cross-section of the beam in the case of ideal alignment. Corresponding radial histograms of rays are displayed for the case φ = 0° (bottom).

Phase retrieval using defocused images of the beam is a well known method, established three decades ago (Roddier & Roddier, 1993 ▸). More general (Paganin & Nugent, 1998 ▸), gradual changes in the three-dimensional (3-D) intensity distribution *I*(**r**) during free-space propagation are related to the phase Φ(**r**) via the transport-of-intensity equation (TIE), 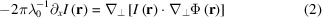
with
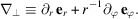
Neglecting the azimuthal component of the transverse gradient 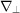, justified by the ‘forgiveness factor’ 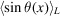 ≃ 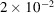 (Urpelainen *et al.*, 2017 ▸), we may derive a simplified correspondence between the direction dir(…) of the Poynting vector **S** and the radial derivative ∂_*r*_Φ(*r*, φ) in parametric dependence on the polar coordinate φ. In analogy to the spherical mirror segment with its – approximately – 1-D focusing properties^2^, the geometrical relation for the wavefront slope reads as 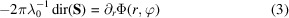
with
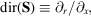
interpreted as the inclination of rays relative to the *x*-axis (Probst *et al.*, 2020a ▸). In practice, this quantity is extracted from two CCD frames of different, sufficiently large defocus ±Δ*x* (with Δ*x* > 0), *i.e.* far outside the focal depth of field (approximately ±2 mm for a blur by 10%) and still beyond the caustic region up to about ±100 mm. To maintain a good signal-to-noise ratio, the integration time is increased to 40 s each, whereas statistical uncertainties are reduced in our non-iterative method by the recording of images at five symmetrical, intra- and extra-focal camera displacements 210 mm ≤ Δ*x* ≤ 250 mm from the nominal focus at *x* = *e* in the coordinates of Fig. 1 ▸. An example within that series – others look similar – is displayed in Fig. 2 ▸.

The central peak, as observed in all defocused CCD images, is used for the coaxial alignment of the ten raw data sets with sub-pixel accuracy, supported by a typical diameter of the order of 10 pixels (FWHM) and a well defined maximum after third-order interpolation. It cannot be explained with low spatial frequency deviations (≲10^−3^ µm^−1^) from the ideal ellipsoidal surface but is rather an effect caused by diffuse wide angle scattering from high spatial frequency errors (≳1 µm^−1^) on the polished mirror shell (Schäfers & Cimino, 2013 ▸), as confirmed qualitatively in 3-D simulations [*Mathematica*/*Optica* (https://www.wolfram.com/mathematica/, https://www.opticasoftware.com)] of an ellipsoid with optional roughness. The ‘spike’ contributes only 1–2% to the total count rate in that off-focal region and is subsequently ‘erased’ from each CCD frame. Across the full image (512 × 512 pixels), the integrated intensity outside the geometrical cross-section of the beam (Fig. 2 ▸) contributes a fraction of ∼33% in the intra- and ∼42% in the extra-focal domain to the total detected flux. However, the differential scatter loss from an inner to the corresponding outer CCD plane is moderate with 11.1 ± 0.7% for the five samples.

**Figure 3:**
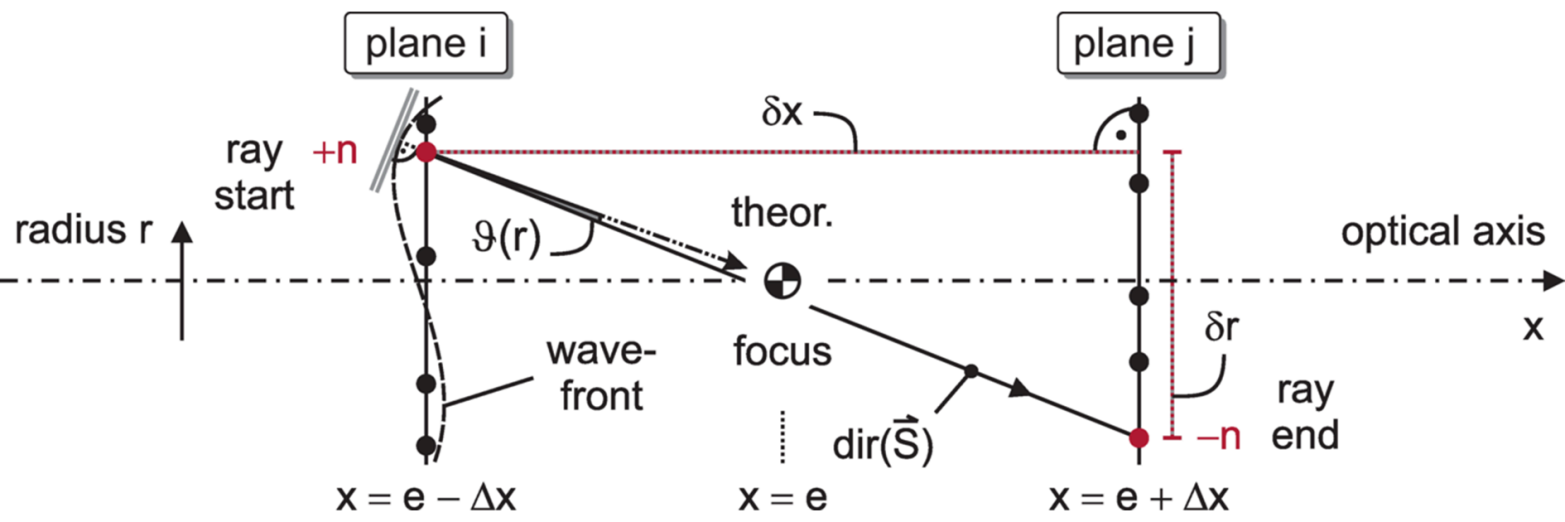
Schematic of the phase retrieval principle. On the left and right of the focus at *x* = *e*, two defocused CCD planes (Fig. 2 ▸) at *x* = *e* ± Δ*x* are indexed by *i* and *j*, respectively. Numbered rays (black dots), whose local density is proportional to *I*(*r*), are traced in a sequential order from the plane *i* to the plane *j*. The direction dir(**S**) = δ*r*/δ*x* of the Poynting vector for the *n*th ray (red) is proportional to the slope 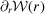, marked by the doubled grey line on the left, and differs from that of the perfect spherical phase by a small angle ϑ(*r*). The integration of this differential slope yields the wavefront error. The graphic is adopted from Probst *et al.* (2020*a* ▸).

The 2-D phase problem of the axisymmetric geometry is reduced to a serial evaluation of 1-D wavefront slopes by taking radial cross-sections *I*(*r*, φ) of the third-order interpolated intensity distribution at an angle 0° ≤ φ < 180° in each CCD plane, as sketched in Fig. 2 ▸, and the bijective mapping 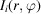

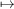

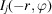
 for *i* ≠ *j* between two of them. An essential constraint of the TIE-based phase retrieval method in general and our implementation in particular requires the conservation of energy along propagation. To compensate a slightly varying (around ±2.1%) power in *I*(*r*, φ) due to scattering and sagittal deflection, the integral 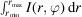 with 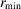 = 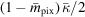, 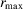 = 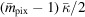 and 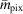 = 512 is re-normalized to the same number of 2^*N*^ rays in all planes. As depicted in Fig. 3 ▸, the continuous intensity distribution is further discretized to a histogram. Each one of the 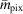 bins of width 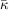 contains a distinct number *f*_*m*_ of rays distributed around the central position 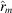 of the *m*th pixel, 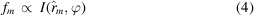
with
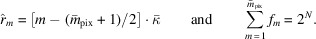
With this convention and the sufficiently large exponent *N* = 17, the radial sampling period is limited by the spatial resolution of the camera, close to 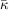 = 13.5 µm. Via 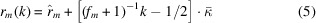
with
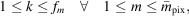
and the rule 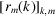

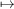

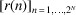
, the histogram is converted from a nested sequence in (*k*, *m*) to a ‘train’ of strictly separated and sorted positions *r*(*n*) in a variable density, representing the intensity *I*(*r*, φ), as illustrated in Fig. 3 ▸. To obtain the direction dir(**S**) of the energy flow (Probst *et al.*, 2020*a* ▸), the difference δ*r*(*n*) ≡ *r*_*j*_(−*n*) − *r*_*i*_(*n*) between start and end point of the *n*th ray in planes *i* and *j*, respectively, is divided by the propagation distance δ*x* ≡ *x*_*j*_ − *x*_*i*_. The numerical values of the projections 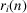

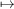

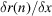
 on the inner (*x* < *e*) and 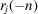

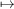

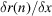
 on the outer (*x* > *e*) planes are tabulated and smoothly fitted to the radial phase slope ∂_*r*_Φ(*r*) ∝ dir(**S**) from equation (3) within the regions of the geometrical beam cross-section (Fig. 3 ▸) by Legendre polynomials up to the 45th order.

With that bidirectional approach, slight mismatches of the CCD recordings in the intra- and extra-focal domain (Fig. 2 ▸), like an excentricity and enlarged scattering for the latter due to technical limitations in our setup, are balanced to a far extent.

## 4. Focus reconstruction and figure error mapping

**Figure 4:**
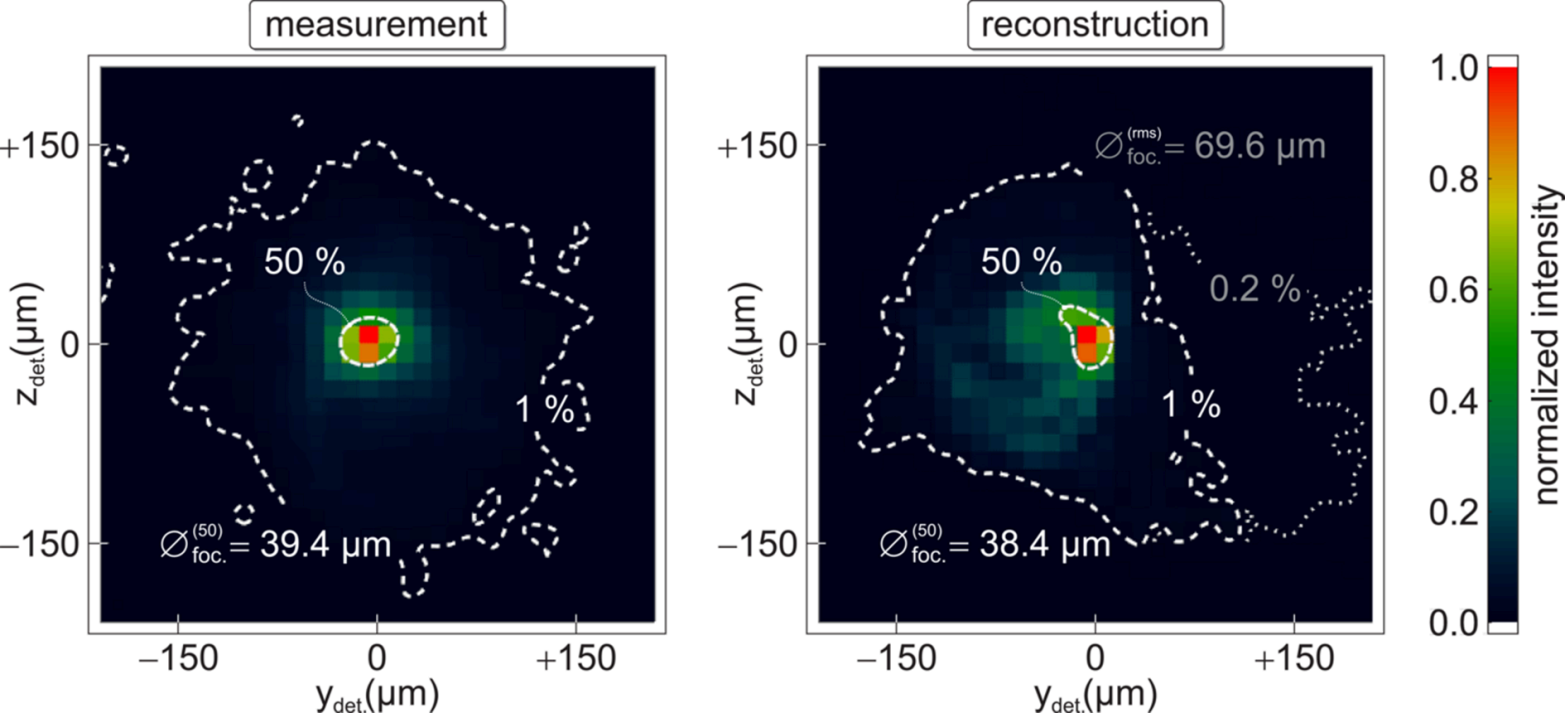
Measured (left) and reconstructed (right) focus at *x* = 553.5 ± 0.6 mm, the latter obtained via propagation of the aberrated wavefront. Both plots are composed of 32 × 32 pixels. White contours indicate intensity levels relative to the third-order interpolated maximum, where the innermost (dashed) refers to 50%. These FWHM values are estimated to ∼39 µm, whereas an r.m.s. spot size of 69.6 µm in the reconstruction is attributed to weak but extended scattering of ∼0.2–1%.

Across the beam cross-section within (*e* − *x*_1_)^−1^Δ*x*(*D*_1_/2) ≤ |*r*| ≤ (*e* − *x*_2_)^−1^Δ*x*(*D*_2_/2), the slope ∂_*r*_Φ(*r*, φ) is evaluated at a step size of ∼3.64 µm in the radial direction and with an increment of 2° in the polar angle 0° ≤ φ < 180°, as sketched in Fig. 2 ▸. Using the vacuum wavenumber 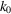

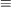

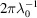
, the normalized 3-D Poynting vector **S**_±_(*r*, φ) for propagation from the plane at *x* = *e* ± Δ*x* to the focus at *x* = *e* then reads as 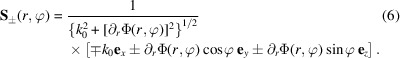
In total, 3.9 × 10^5^ rays are traced from the five inner and corresponding five outer planes to the focus, whose reconstructed position is located at *x* = 503.5 ± 0.6 mm by an internal algorithm of the software (*Optica*), based on the criterion of a minimized spot size of 69.6 µm (r.m.s.). In Fig. 4 ▸, the experimental result is compared in the pixel matrix with that simulated focal spot, whose asymmetry on an intensity level of ∼10–30% and minor but widespread scattering (0.2%) can be ascribed to an accidental inaccuracy during the measurements, as noted at the end of Section 3. Nevertheless, a third-order interpolation allows the 2-D averaged FWHM (50%) of both to be estimated, and we find them in good agreement with 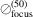 = (38.9 ± 0.5) µm.

**Figure 5:**
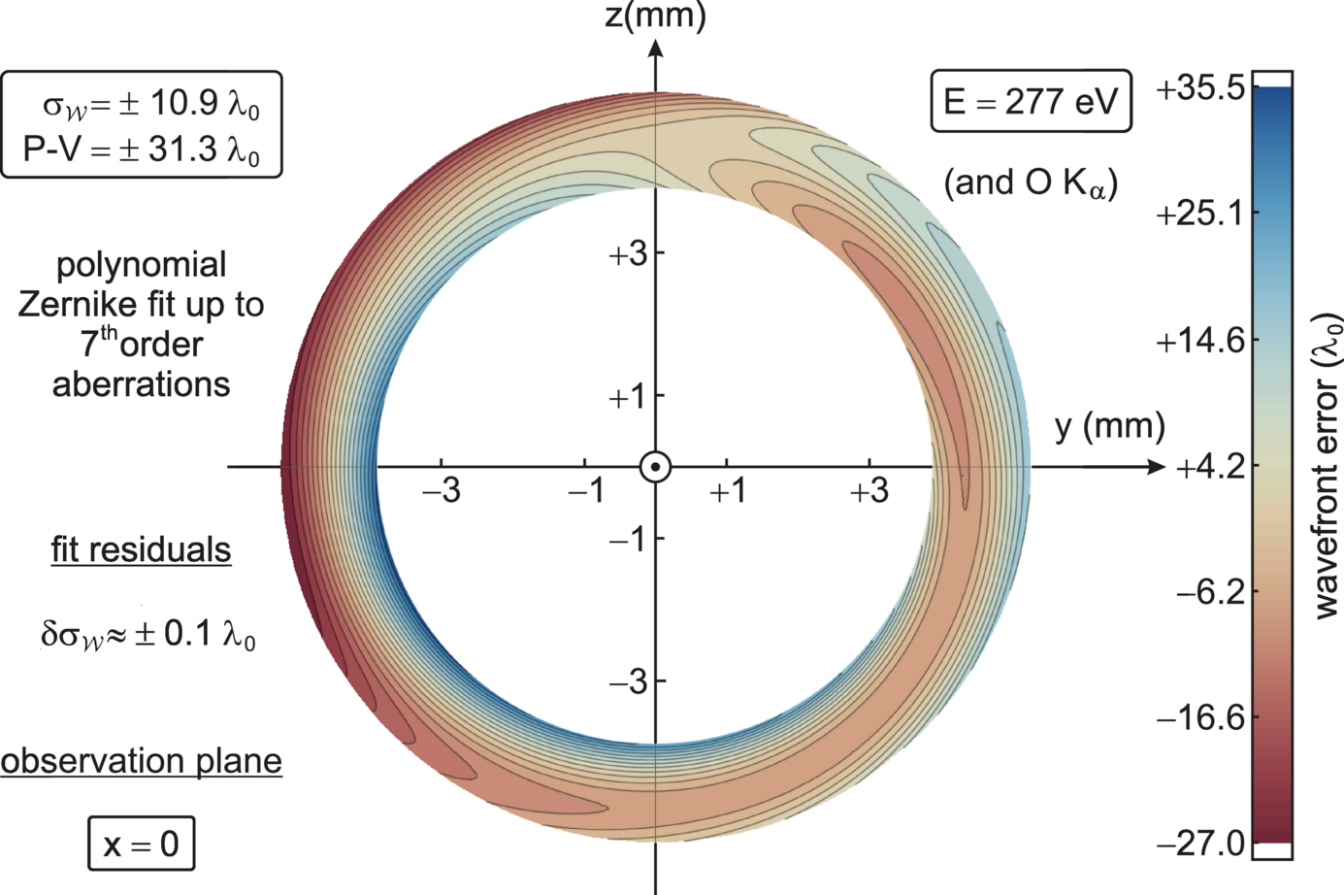
Retrieved wavefront error 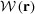 on the annular cross-section of the beam at *x* = 0, *i.e.* in the optical centre of the ellipsoid (Fig. 1 ▸), in units of λ_0_ at 277 eV. Five different CCD image pairs within 210 mm ≤ Δ*x* ≤ 250 mm are analysed with the algorithm (Figs. 2 ▸ and 3 ▸). The averaged data are fitted by means of a Zernike expansion (Fig. 6 ▸) up to seventh-order aberrations, with an r.m.s. value 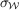 = 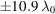. See also the movie in the supporting information.

**Figure 6:**
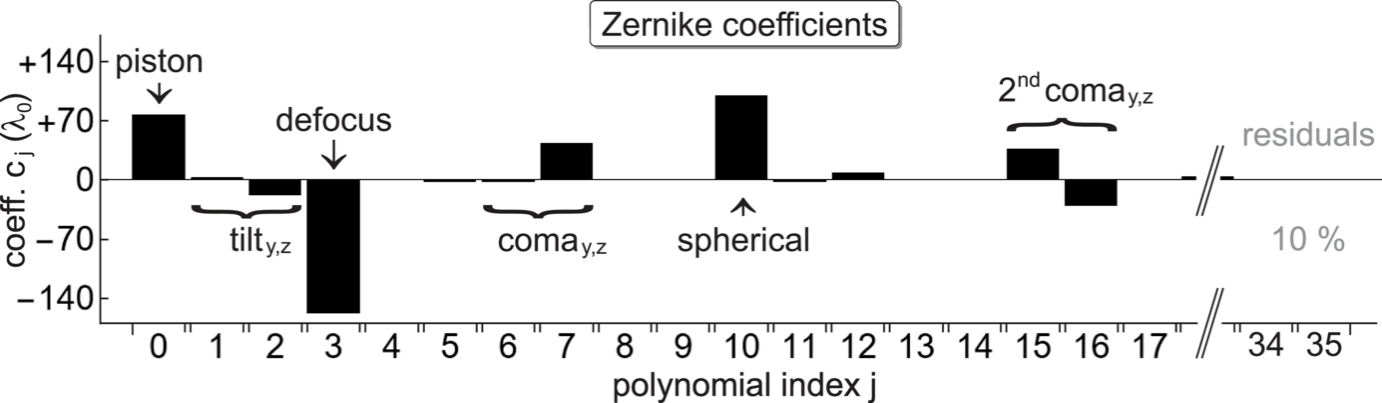
Coefficients *c*_*j*_ in the polynomial Zernike expansion 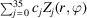 of the wavefront error from Fig. 5 ▸ up to seventh-order aberrations. The numerical values are given in units of λ_0_ at 277 eV. A few selected terms like defocus or coma are labelled.

To extract the deviation of the real phase Φ(*r*) from that of an ideal sphere at a given angle φ in the defocused plane at *x* = *e* ± Δ*x*, we define the differential slope 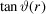

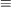

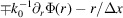
 in the radial direction (Fig. 3 ▸), again confined to the annular region of the geometrical beam cross-section, as specified in the context of equation (6). The wavefront error 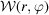 in units of λ_0_ then follows from an integration along *r*, 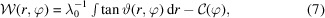
where
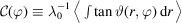
denotes the arithmetic mean of the integral within its radial domain of definition, representing the constant for piston correction.^3^ Computed for 0° ≤ φ < 180°, each one of the 5 × 2 sample sets (five displacements ± Δ*x* in a bidirectional approach) is projected by central dilation to a common plane, *e.g.* the symmetric cut at *x* = 0, and the averaged data for 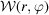 are fitted by an expansion 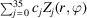 of Zernike polynomials up to their seventh order. The result is shown in the Cartesian coordinate system of Fig. 5 ▸ as a view along −**e**_*x*_, from the focus to the source. In agreement with the common definition, an error 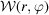 > 0 stands for a wavefront running in advance relative to the ideal sphere. According to Fig. 6 ▸, the fit is characterized by major contributions from piston, defocus and spherical aberration, which compensate each other to some degree. Besides, angle-dependent aberrations up to the fifth Zernike order like tilt and primary as well as secondary coma play a significant role. Coefficients *c*_*j*_ with *j* ≥ 21 for sixth- (and higher) order aberrations add up to no more than 10% of the total wavefront error magnitude and can be neglected approximately. At a standard deviation of ±1.2 λ_0_, the raw data from the 5 × 2 plane-to-plane samples are distributed statistically around the fit (Fig. 5 ▸) with a peak-to-valley (P-V) range of ±31.3 λ_0_ and an r.m.s. value 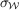 = 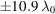, whose uncertainty is estimated to 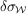

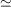

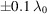
. In terms of the ‘coefficient of determination’, *R*^2^ = 98.8% indicates a good accuracy of the modelled wavefront shape, where not only the similarity of all 5 × 2 evaluated samples (‘reproducibility’) but also the slow variation of 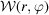 in both variables (‘smoothness’) support the quality of the Zernike fit.

**Figure 7:**
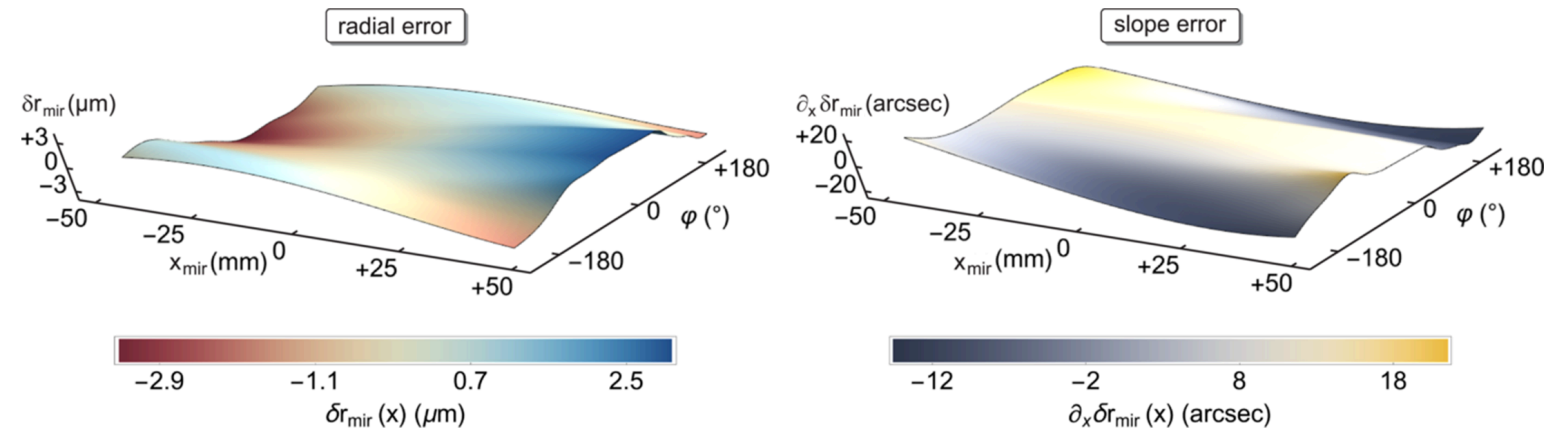
Radial, *i.e.* combined figure/alignment error δ*r*_mir_(*x*, φ) on the left and slope error ∂_*x*_δ*r*_mir_(*x*, φ) on the right along surface lines of constant φ, calculated using equation (8). The axial position *x*_mir_ is measured relative to the geometrical centre of the mirror, whereas the angle φ refers to the polar orientation as defined in Fig. 2 ▸.

In the range 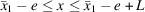, as sketched in Fig. 1 ▸, the wavefront error 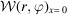 from Fig. 5 ▸ is mapped (Siewert *et al.*, 2012 ▸; Barty *et al.*, 2009 ▸) as the surface deviation 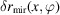

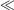

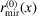
 onto the nominal ellipsoid from equation (1) via 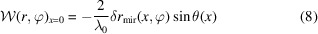
for
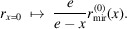
From the geometry, we derive 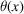 = 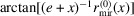 − 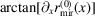 for the grazing angle of total external reflection. That unbiased^4^ figure and alignment error δ*r*_mir_(*x*, φ) varies within an amplitude of ±3.20 µm (P-V), as depicted in Fig. 7 ▸ (left). Averaged over the full mirror surface, we find 〈δ*r*_mir_(*x*, φ)〉 = ±1.14 µm (r.m.s.).

At least in the regime of geometrical optics, however, the focusing capabilities are determined primarily by the slope error ∂_*x*_δ*r*_mir_(*x*, φ) along the *x*-axis, *i.e.* the derivative of the radial profile irregularity in the direction of beam propagation. Fig. 7 ▸ visualizes on the right the tangential slope, characterized on average by 〈∂_*x*_δ*r*_mir_(*x*, φ)〉 = ±8.8 arcsec (r.m.s.) and a P-V of ±18.1 arcsec. The polar component is much smaller, around ±0.5 arcsec (r.m.s.), and contributes less to aberrations.

Based on this information, the simulation of the mirror with its perturbed radius 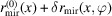 reveals a focal spot size of 63.9 µm (r.m.s.) at *x* = 504.1 mm, close to the result from the direct reconstruction using equation (6). On the other hand, the mirror can be modelled as a reflective but ‘wavy’ surface whose slope error leads to a simulated focus diameter that coincides with measurements within a tolerance of 9%.

## 5. Conclusion

Soft X-ray wavefront sensing at an axisymmetric, ellipsoidal mirror with an effective angular acceptance of 1.7 millisterad and a focal distance of 1 m is demonstrated in a table-top experiment using C *K*_α_ fluorescence (277 eV), based on a measurement of the 3-D intensity distribution under defocus and a novel algorithm for its analysis.

Since the full (512 × 512) pixel matrix of the CCD camera can be used to probe the beam and each wavefront data set is sampled by (4.6 ± 0.6) × 10^4^ pixels therefrom, our method combines high spatial resolution and sensitivity. The latter is of particular relevance for low-flux sources or synchrotron beamlines, providing a moderate count rate of, for example, ∼4.3 × 10^5^ s^−1^ like in our laboratory setup. The technique requires minimal experimental and instrumental effort, only two recordings by a 2-D CCD camera on a linear translation stage, and works in the regime of a limited transverse coherence length which is estimated to ∼50 pixels in the focal plane for the micro-fluorescence source (2 µm) in use. Customized ray tracing is applied to develop an optimized, robust code^5^ in a noisy environment for the reproducible retrieval of an even large wavefront error of ±10.9 λ_0_ (r.m.s.) or more at optical elements of rotational symmetry, *i.e.* the piston-corrected, relative aberration is 2-D resolved in polar coordinates (*r*, φ). As an additional benefit, the wavefront information allows the focus to be reconstructed, whose spot size of ≲39 µm (FWHM) and position (0.35% off the nominal focal distance) nearly coincide with the experiment and theoretical expectation. The wavefront distortion is moreover mapped onto the surface of the ellipsoid as its combined figure and alignment error at a step size of ∼400 µm in the axial (*x*) and ∼250 µm in the polar (φ) direction. The average of ±1.1 µm (r.m.s.) corresponds to a slope error of ±8.8 arcsec (r.m.s.). In a closed loop, the full system with that distorted mirror is simulated in a Monte Carlo ray tracing routine (*Optica*), and the outcomes confirm the findings above.

However, the mirror as considered in this work was known for its above-average microroughness and scatter, according to information from the manufacturer. In future, better polished samples should thus ease the data analysis and yield more accurate results. Furthermore, our proof-of-principle experiment suffered partially from an accidental shift, tilt or vibrations which are supposed to induce an additional perturbation to the phase especially in the extra-focal half-space.

Besides gimbal mounting for the optic under test to reduce misalignment, mechanical components with a tolerance of ±10 µm along the optical *x*-axis and ±0.1 µm in the *y*, *z*-direction should be employed in future for precise in-line measurements with the moving camera. In consequence of a good adjustment, the figure error of the optic can be isolated, and the wavefront is expected to be determined at an enhanced accuracy well below the Maréchal criterion (Probst *et al.*, 2020*a* ▸). To simplify the image pre-processing, a clear, unique criterion for definition of the optical axis and for centring of the CCD frames to the exit pupil must be specified (Ruiz-Lopez *et al.*, 2020 ▸). To shorten the integration time of 40 s per image, the source flux may be enhanced and stray light should be lowered, preserving a high signal-to-noise ratio in near photon-limited detection.

Possibly, the phase retrieval scheme might be extended to the absolute metrology mode (Frith *et al.*, 2023 ▸). In the code, the compromise between dynamic range and resolution of the wavefront sensor is expressed by the density of rays, which is set to 256 on average per pixel presently. In future versions of the program, this crucial quantity should be adapted to demands on the P-V range and the uncertainty of the r.m.s. wavefront error, for instance. As a mid-term goal, the algorithm (*Mathematica*) shall be evolved to a fast routine (Python *etc*.), such that quick auto-alignment and quality control of axisymmetric X-ray optics, including zone plates, lenses and parabolic or Wolter-type mirrors, becomes feasible in the laboratory and at large-scale facilities like synchrotrons or free-electron lasers (Frith *et al.*, 2023 ▸). In a final step, an adapted reflective/diffractive wavefront corrector (Probst *et al.*, 2023 ▸) – as the low-absorption soft X-ray alternative to the refractive phase plate (Dhamgaye *et al.*, 2020 ▸) – may be designed, fabricated and applied, to compensate for the mirror’s imperfections.

## Supplementary Material

Supporting information file. DOI: 10.1107/S1600577524003643/ys5105sup1.mp4

## Figures and Tables

**Table 1:** Geometrical design parameters of the ellipsoidal mirror shell, as defined in Fig. 1 ▸

*a*	*b*	*L*	*D*_1_	*D*_2_	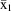	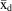	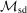
500.077 mm	8.75475 mm	100 mm	13 mm	15.46 mm	165 mm	1000 mm	3.65

**Table 2:** Sine wave test of the algorithm at various amplitudes and spatial frequencies

	Amplitude (±)					
	3 × 10^−6^ nm	1 nm	10 nm	100 nm	1000 nm	3 × 10^3^ nm
Spatial frequency	80 m^−1^	40 m^−1^	20 m^−1^	10 m^−1^	5 m^−1^	5 m^−1^
Residuals (r.m.s.)	0.5%	0.7%	1.2%	2.4%	4.6%	7.7%

## Footnotes

1 Within this text, the wavefront and its error, if given in units of λ_0_, presume pure C *K*_α_ for simplicity.

2 In Appendix *A[App appa]*, the validity of the phase retrieval code is investigated with respect to this analogy.

3 Any bias does not distort the wavefront, and its subtraction (for *r* < 0 and *r* > 0 separately) corresponds to an offset-free figure error δ*r*_mir_(*x*, φ) around the ideal mirror shape  in equation (8).

4 Equation (7) implies a near-zero mean of δ*r*_mir_(*x*, φ) relative to  along each surface line (φ = const).

5 The *Mathematica*/*Optica* code is available for free from the correspondence author on request.

## Appendix A Validity of the phase retrieval code

The physics behind our phase retrieval method and the basic structure of its algorithmic implementation are identical to that described by Probst *et al.* (2020*a* ▸), where the validity was verified by means of a spherical mirror whose 1-D figure error in the beam direction has been measured independently using the Nanometre Optical Component Measuring Machine (NOM) at Helmholtz-Zentrum Berlin. Our wavefront reconstruction confirmed the NOM reference with an accuracy of ±λ_0_/25 (r.m.s.). The present project adopts and extends this approach toward axisymmetric mirror shells in terms of radial, quasi 1-D wavefront reconstructions along the mirror’s surface lines.

Notwithstanding the conceptual equivalence to Probst *et al.* (2020*a* ▸), in view of an increased complexity compared with the spherical mirror, and regarding the fact that the amplitudes of wavefront (±30λ_0_, P-V) and figure error (±3 µm, P-V) for the ellipsoidal mirror are up to 10^2^× enlarged, functionality and accuracy of the proposed extension of our phase retrieval method to axisymmetric geometries must be verified at known, simple optics. To reduce the computational payload, we simulate an analogue system in terms of a radial 1-D cross-section of the ellipsoid, whose predefined figure error is modelled as a sine or cosine wave^6^ of variable amplitude and spatial frequency in the *x*-direction. The intersection points of the propagated rays with two planes (*i.e.* axial positions of the CCD camera) in a defocus ±Δ*x* then yield the intensity distributions *I*(*r*) which can be analysed like the actual experiment, using the algorithm above. The data in Table 2 ▸ span several magnitudes of figure error amplitudes. Higher amplitudes arise at lower spatial frequencies, qualitatively imitating the typical power spectral density of a real surface. At an amplitude of ±3 µm, roughly equal to the maximal deformation as detected in the measurement (Fig. 7 ▸), the halved sine/cosine wave is reconstructed within a relative standard deviation of 7.7% (r.m.s.). Toward diminished amplitudes in the nanometre regime, several periods along the mirror length are well reproduced, represented by residuals ∼1%, and the code would still work reliably down to the level of machine precision on the femtometre scale.

In practise, the lower limit is set by the CCD resolution, ∼10^−2^–10^−1^ pixels for the interpolated, ‘spiky’ intensity distribution. At a plane-to-plane propagation distance of 460 ± 40 mm, this resolution is translated into an angular accuracy around ±6.1 × (10^−2^–10^−1^) arcsec. Since the experimental slope of ±8.8 arcsec (r.m.s.) corresponds to a radial error (Fig. 7 ▸) of ±1.14 µm (r.m.s.) and due to the linearity of integration in equation (7), the lower limit for the given surface profile is estimated to ±8–80 nm or, using equation (8), a wavefront accuracy of ±8 × (10^−2^–10^−1^)λ_0_ (r.m.s.). The latter values are comparable with the uncertainty  (r.m.s.) of the mean wavefront error and the standard deviation within the raw data set of 5 × 2 evaluations (Section 4), respectively.

In all, the sine wave test illustrates and confirms the expectation that ‘our’ sensor, like most others, works best for smooth intensity distributions and at small amplitudes δ*r*_mir_(*x*, φ), corresponding to wavefront errors (P-V) up to a few λ_0_ as aimed for in the future.
